# Antibodies to the Muscarinic Acetylcholine Receptor M3 in Primary Biliary Cholangitis Inhibit Receptor Function on Cholangiocytes

**DOI:** 10.3389/fimmu.2020.01151

**Published:** 2020-06-30

**Authors:** Christian Mayer, Beate Preuss, Julia Grottenthaler, Christoph Berg, Reinhild Klein

**Affiliations:** ^1^Department of Internal Medicine II, University of Tuebingen, Tübingen, Germany; ^2^Department of Internal Medicine I, University of Tuebingen, Tübingen, Germany

**Keywords:** primary biliary cholangitis, functional autoantibodies, muscarinic acetylcholine receptor 3, cholangiocytes, Chinese hamster ovary cells, disease activity

## Abstract

**Background and Aims:** In primary biliary cholangitis (PBC), antibodies to a peptide of the muscarinic acetylcholine receptor 3 (mAChR3) have been described. Since the mAChR3 is expressed on cholangiocytes and mAChR3-signaling is involved in the pathogenesis of chronic inflammatory biliary diseases, we wanted to investigate whether anti-mAChR3-antibodies influence the function of the receptor and the proliferative response of cholangiocytes.

**Methods:** Immunoglobulins were isolated by ammonium sulfate precipitation using sera from patients with PBC (*n* = 63) and with other chronic liver disorders (*n* = 150). All immunoglobulins were analyzed by a luminometric assay using Chinese hamster ovary (CHO) cells overexpressing the mAChR3 and cholangiocytes (TFK-1-cells) expressing the receptor constitutively. Cell proliferation was measured by ^3^H-thymidine assay. PBC patients were also analyzed in the follow-up.

**Results:** Antibodies inhibiting the mAChR3 were found in 49 and 79% of PBC patients using CHO-cells or TFK-1-cells, respectively, but only in up to 26% of controls (*p* < 0.01). Stimulatory antibodies were hardly detected. Antibody reactivity only marginally changed during the course of the disease, independently of the choice of treatment (ursodeoxycholic acid, immunosuppressive therapy, or no medication). There was no correlation with laboratory, clinical or histological parameters, but the antibodies were more frequently found in PBC patients with a benign course (96%) than in patients with active disease progressing to late stages within 10 years (57%; *p* < 0.01). Proliferation of cells was not influenced by immunoglobulins from PBC-patients.

**Conclusion:** Sera from patients with PBC contain inhibitory antibodies to the mAChR3 on cholangiocytes (TFK-1 cells) without influencing TFK-1-cell proliferation. These antibodies were predominantly observed in patients with non-progressing PBC.

## Introduction

Functional autoantibodies interacting with receptors have been reported in several organ-specific autoimmune disorders, such as Graves' disease, myasthenia gravis, or idiopathic cardiomyopathy (1–3). In other autoimmune disorders, diagnostically highly relevant antibodies occur, but mostly without organ specificity or functional activity, as shown for antinuclear antibodies in collagen disorders or several types of autoantibodies in different autoimmune liver diseases. Interestingly, in recent years, it has emerged that functional antibodies can also occur in those disorders, which may help to explain at least some of their specific clinical symptoms. For instance, in patients with primary Sjoegren syndrome (pSS) autoantibodies to the muscarinic acetylcholine receptors, especially of the M3-type (mAChR3) have been described (4–6). These receptors are expressed on the surface of salivary acinar glands (5, 7) and belong to the G protein-coupled receptors (GPCR) (8). There is now growing evidence that perturbation of muscarinic receptor function by the presence of those antibodies accounts in large part for the glandular hypofunction and are also responsible for some of the extraglandular features of pSS (4, 9–16). Also, in systemic sclerosis autoantibodies, which inhibit the muscarinic transmission, may be responsible for gastrointestinal dysmotility (17). Both disorders can be associated with autoimmune liver disorders, especially primary biliary cholangitis (PBC) (18, 19). In this respect, it is of interest that the mAChR3 is expressed on cholangiocytes regulating their regeneration and proliferation, but not on hepatocytes (20). Twenty-five years ago, antibodies to the nicotinic acetylcholine receptor had already been described in PBC (21, 22). From preliminary studies using a peptide of the mAChR3, we had evidence that also antibodies to the mAChR3 seem to be present in this disease (23–25). Moreover, using human cell lines, we could confirm that the mAChR3 is constitutively expressed by cholangiocytes (TFK-1 cells), but not hepatocytes (HepG2 cells) (26).

Antibodies to mAChR3 have been demonstrated by different methods. The “gold standard” for the detection of functionally active antibodies has been bioassays using the inhibition of smooth muscle from bladder or colon as detection system (10, 16, 27–29). Several other methods, including pharmacological assays, have been established in further studies [for literature review see (30)]. However, the application of bioassays to large populations is limited for several reasons (25); therefore, immunodominant epitopes within the mAChR3 have been identified and applied in enzyme-linked immunosorbent assays (ELISA). In PBC, antibodies to several loops of this receptor have been found (31). However, it soon became evident that the functional antibodies are directed against conformational epitopes, so that no correlation between bioassays and assays using linear epitopes or recombinant antigens was observed (32–34).

We have, therefore, recently established a novel test system for the demonstration of functional anti-mAChR3-antibodies in patients' sera. It is based on the determination of downstream signaling of mAChR3 using Chinese hamster ovarian (CHO) cells transfected with plasmids encoding mAChR3 and a green fluorescence protein (GFP)/aequorin fusion protein (30). Thus, activation of G protein-coupled receptors (GPCR) leads to an opening of Ca^2+^ channels in the endoplasmic reticulum; the resulting efflux of Ca^2+^ can be visualized with a luminometric assay. This test system produces specific and reproducible results, and we could confirm the high prevalence of functional anti-mAChR3 antibodies in pSS (30). Preliminary data indicated that they can be detected also in sera from PBC patients (25). The aim of the present study was, therefore, to investigate the presence of these antibodies in more detail in patients with cholestatic autoimmune liver disorders using mAChR3-expressing CHO-cells, but also cholangiocytes constitutively expressing the mAChR3. Moreover, we wanted to see whether the antibody reactivity is influenced by patients' treatment and whether the antibodies have an effect on cell proliferation.

## Patients

Sera from 63 patients with clinically, histologically, and serologically defined PBC (56 females, mean age 50 years, range 21–92 years; 7 males, mean age 53 years, range 44–60 years) were analyzed. In all patients, diagnosis had been proven by histology and/or typical clinical and laboratory parameters. PBC patients were all positive for PBC-specific antibodies [antimitochondrial antibodies reacting with the M2-antigen/pyruvate-dehydrogenase complex-E2 (PDC-E2), antinuclear antibodies to nuclear dots (sp100), and nuclear membrane (gp210)] (35).

Sera from 38 of the 63 patients with PBC were further investigated during the course of the disease. Thirty-six of these 38 patients were anti-M2 positive, one had only antibodies to nuclear dots (sp100), and one anti-gp210 antibodies. Thirty-one patients were in stage I/II of the disease at time of first investigation, seven were in stage III/IV.

Four of the thirty-eight patients remained without any therapy for a follow-up of 9–177 months (median 103 months); 18 patients received ursodeoxycholic acid (UDCA) for 12–206 months (median 121 months); and 16 patients were treated with UDCA and immunosuppressive therapy due to association with other autoimmune disorders, increased disease activity, or orthotopic liver transplantation (OLT; *n* = 5). All of them received glucocorticoids, five were additionally treated with azathioprine, four with cyclosporine, three with methotrexate, two with tacrolimus, and two with mycophenolate-mofetil (follow-up 11–213 months, median 108 months).

The 38 patients were divided into two groups according to their clinical course (progressive vs. non-progressive): Patients who were in late stages at time of first diagnosis or who were in stage I/II but developed signs of liver cirrhosis within 5–10 years (histologically, development of stage III/IV, hyperbilirubinemia, portal hypertension, necessity of liver transplantation, and death because of liver failure) were assigned to the progressive group (*n* = 24); patients who were in stage I/II at first diagnosis and did not develop any signs of disease progression for at least 5–10 years were assigned to the non-progressive group (*n* = 14).

As controls, sera from 50 patients with primary sclerosing cholangitis (PSC) (proven by endoscopic retrograde or magnetic resonance cholangio-pancreaticography; 22 females, mean age 43 years, range 19–72 years; 28 males, mean age 33 years, range 19–59 years), from 50 patients with viral hepatitis B or C (22 females, mean age 42 years, range 22–60 years; 28 males, mean age 38 years, range 16–63 years), from 50 patients with alcoholic liver disease (ALD) (15 females, mean age 51 years, range 34–63 years; 35 males, mean age 53 years, range 25–72 years), and from 50 healthy blood donors (26 females, mean age 41 years, range 20–62 years; 24 males, mean age 30 years, range 18–61 years) were investigated.

All patients had been seen by one of the authors (CB or JG) and had given their informed consent to participate in the study. The healthy controls were derived from students or blood donors (kindly provided by Dr. D. Wernet, Institute for Transfusion medicine, Tuebingen).

The study was approved by the local ethics committee and was performed in accordance with the Helsinki declaration. All patients gave written informed consent.

## Materials and Methods

### Purification of Immunoglobulins From Patients' Sera

Immunoglobulins were isolated from patients' sera by ammonium sulfate precipitation as described (26). This method was chosen because we have shown that it gives more reliable results than immunoglobulins purified by Melon IgG Spin purification kit (26). The immunoglobulins were used at a final dilution of 1:100 (corresponding to about 0.15–0.17 mg protein/ml). The optimal dilution of the proteins had been determined in dilution studies (data not shown) (26).

**Figure 1:**
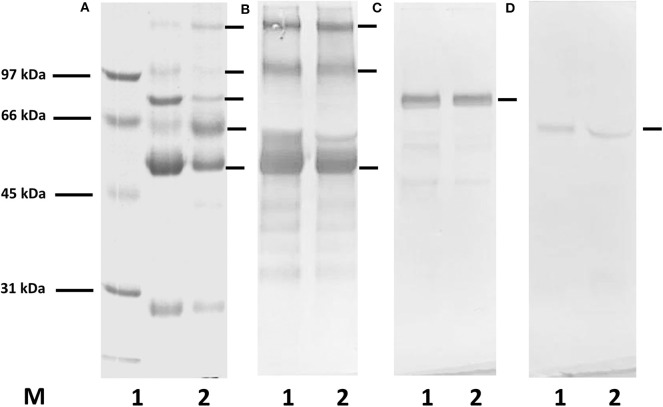
SDS-gel electrophoresis and Western blotting for the demonstration of proteins in the immunoglobulin fractions isolated by Melon IgG Spin purification kit and ammonium sulfate (AS) precipitation from a serum of a healthy donor. **(A)** Coomassie staining, **(B–D)** Western blotting with anti-human HRP-conjugated antibodies: **(B)** anti-human IgG, **(C)** anti-human IgM, **(D)** anti-human IgA antibodies; M, molecular weight marker; lane 1, immunoglobulin purified from serum using Melon IgG purification kit; lane 2, AS precipitated proteins. In both fractions only proteins related to immunoglobulins can be observed.

The purity of the immunoglobulin fraction obtained by ammonium sulfate precipitation of patients' sera was analyzed by SDS-gel electrophoresis and Western blotting (Figure 1). All protein bands in the fractions visualized by Coomassie staining in the gels could be attributed to IgG, IgM, or IgA, and no further proteins were detected with this method.

In order to see whether the residual ammonium sulfate present in the immunoglobulin preparation after precipitation might influence the functional assay, the precipitated proteins from patients' sera were dialyzed against Hanks' balanced salt solution (HBSS) and compared with the results obtained with non-dialyzed probes (proteins added 24 h after transfection). There was only a marginal effect, and therefore, we used non-dialyzed immunoglobulin fractions in further studies (26). Moreover, we used the ammonium sulfate solution itself without patients' immunoglobulins, and it also did not affect the functional assay.

### Demonstration of PBC-Specific Antibodies

Patients' sera and immunoglobulins were tested for anti-M2 antibodies by an in-house enzyme-linked immunosorbent assay (ELISA), as described (36, 37), using the M2-antigen prepared from beef heart mitochondria, as well as the recombinant E2-subunit of the pyruvate dehydrogenase complex (PDC-E2) of the 2-oxoacid dehydrogenase complex (OADC) and the branched-chain-2 oxoacid dehydrogenase complex (BCOADC) (Diarect, Freiburg, Germany).

Furthermore, sera and immunoglobulins were tested by immunofluorescence test using Chang-liver cells for the demonstration of PBC-specific antinuclear antibodies (antibodies to nuclear dots/sp100, antibodies to nuclear membrane/gp210, antibodies to centromeres) (36, 37).

### Cell Lines

CHO cells (CHO-K1) stably transfected with a calcium-sensitive bioluminescent fusion protein consisting of aequorin and green fluorescent protein (GFP) were kindly provided by Dr. Stefan Offermanns (Max-Planck-Institute for Heart and Lung Research, Bad Nauheim, Germany) and were cultured as described (30). These cells are indicated as CHO/G5A.

TFK-1-cells (ACC-344) originating from bile duct carcinoma (obtained from Leibniz DSMZ-German Collection of Microorganisms and Cell Cultures, Braunschweig, Germany) were cultured in RPMI medium (Biochrom GmbH, Berlin, Germany), supplemented with 10% (v/v) fetal bovine serum (Gibco, Life Technologies, Darmstadt, Germany), and 160 μg/ml Gentamycin (Merck-Sigma-Aldrich, Munich, Germany).

Cells were maintained at 37°C in a humidified atmosphere of 5% CO_2_ and used between passages 8 and 20.

### Transfection

Transfection of GFP/aequorin-transfected CHO-K1 cells with mAChR3 plasmid DNA was performed as recently described (30).

TFK-1-cells constitutively expressing mAChR3 (26) were transiently transfected with 1 μg/ml of a calcium-sensitive fusion protein consisting of aequorin and GFP (38) using FuGENE 6 transfection reagent (Promega, Madison, WI, USA), according to manufacturer's protocol. Expression vector plasmid and transfection reagent were diluted in RPMI serum-free medium, and a FuGENE 6 to DNA ratio of 3:1.

Transfection efficiency was proven microscopically by evaluating the green fluorescence signal.

### Analysis of the Effect of Patients' Immunoglobulins on the mAChR3 Function

The effect of patients' immunoglobulins on the mAChR3 function was analyzed as previously described (30). Briefly, mAChR3 transfected CHO/G5A cells were used in the luminometric assay. Ammonium-sulfate precipitated immunoglobulins from patients' sera were added to the cells for 1 h in a dilution of 1:100 (0.15–0.17 mg immunoglobulins/ml), which had been shown in previous studies to give optimal results (30).

For a measurement of the effect of immunoglobulins on TFK-1 cells, the method was slightly modified. Thus, transfected cells were seeded in 96-well plates at 12,000 cells per well and incubated overnight to a confluence of 80–90%. The optimal concentration of cells had been determined prior to the study (26). Culture medium was removed, and cells were pre-incubated with Coelenterazine h in HBSS without Ca^2+^ for 1 h at 37°C. Then, cells were incubated for 1 h in HBSS containing 2 mM CaCl_2_. Ammonium-sulfate-precipitated immunoglobulins from patients' sera were added as quadruplicates to the cells for 1 h in a dilution of 1:100. As controls, untreated transfected and non-transfected cells were used.

After addition of the mAChR3-agonist carbachol (2 μM; Sigma-Aldrich, Steinheim, Germany) to the CHO- and TFK-1 cells, respectively, the change in intracellular calcium which results in emitted light was measured during an integration interval of 20 s by a 2460 MicroBeta2 LumiJet luminometer (Perkin Elmer, Downers Grove, IL, USA). Results are given as percentage of relative luminescence units (RLUs) of cells without immunoglobulins (% of RLUs [RLUs w/o incubation with Ig = 100%]). According to the analysis of immunoglobulins from healthy controls and evaluation by ROC curves, stimulation of the mAChR3 is defined for CHO- and TFK-cells as values ≥130% and inhibition as values ≤70%.

### ^3^H-Thymidine Proliferation Test

To investigate the influence of patients' immunoglobulins on cell proliferation the ^3^H-thymidine incorporation assay was used.

CHO-G5A cells transfected with the mAChR3 and TFK-1-cells constitutively expressing the mAChR3 were seeded into 96-well plates (0.8–1 × 10^4^ cells/100 μl) and incubated in a humidified atmosphere containing 5% CO_2_ at 37°C until a confluence of 80–90% was reached. Immunoglobulins from patients' sera were added at a dilution of 1:100 for another 24 h; afterwards, 20 μl ^3^H-thymidine solution (0.4 μCi or 14.8 kBq) per well was added for 16 h. Thus, the cells were incubated with patients' sera in total for approximately 40 h. Thereupon, supernatants were discarded and plates were washed three times with 50 μl/well of distilled water. Plates were air dried, and then 25 μl scintillation fluid was added to each well. Thymidine uptake was measured in counts per minute (cpm) by a MicroBeta2 LumiJet with factory settings of Perkin Elmer. Cell proliferation was classified in stimulation, inhibition, and no effect, analogous to the functional mAChR3 assay. The ^3^H-thymidine uptake of untreated cells served as negative control. Results are given as a percentage of cpm obtained with cells w/o (with/without) immunoglobulins.

According to the analysis of immunoglobulins from healthy controls, stimulation of cell proliferation is defined for CHO- and TFK-cells as values ≥130%, and inhibition as values ≤70%.

### Statistical Analyses

All experiments were performed in quadruplicates, and tests were repeated a minimum of three times. Outliers were analyzed by the Dixon's test.

Results of the luminometric assay are given as % RLU (percent of RLUs with immunoglobulins of RLU obtained without immunoglobulins), of the proliferation test as percent of cpm obtained with cells w/o (with/without) immunoglobulins.

Normal values for the functional assays had been calculated by receiver operation curves (ROC) comparing the reactivity obtained with immunoglobulins from patients with that of disease and healthy controls aiming at a specificity of 90–95% resulting in a “normal range” between 70 and 130%, i.e., immunoglobulins showing in this assay values ≤70% were considered to have inhibitory, those with values ≥130% stimulatory antibodies (30).

Statistical analysis was carried out using GraphPad Prism 6.0. All data are expressed as individual values and median. The unpaired Mann-Whitney *U*-test was used for comparison of antibody reactivity between patient groups, and Wilcoxon's test was used for paired data. Prevalence was compared using Fisher's exact test. A *P* < 0.05 was considered statistically significant.

## Results

### Effect of Immunoglobulins From Patients With Liver Disorders on mAChR3 Activity Using mAChR3 Overexpressing CHO/G5A-Cells

**Figure 2:**
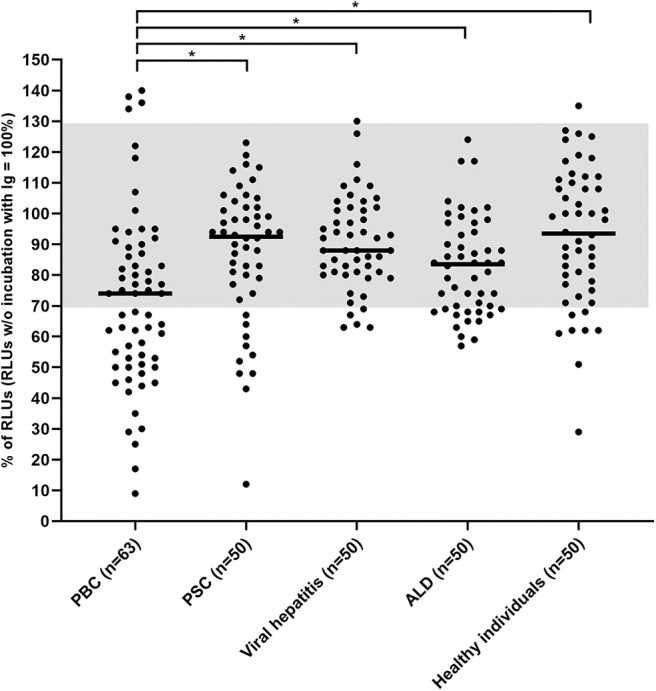
Carbachol-induced [Ca^2+^] signal in the functional assay after 1 h incubation of mAChR3 transfected CHO/G5A cells with immunoglobulins from patients' sera. Data are given as percentage of the results w/o incubation. Each data point represents the mean of four independently performed experiments per patient. –, median; gray, normal range; *significant as compared to PBC (*P* < 0.005).

Using CHO/G5A-cells overexpressing the mAChR3, the carbachol-induced [Ca^2+^] signal was significantly lower with immunoglobulins from PBC-patients as compared to healthy individuals and the other groups of patients with chronic liver disorders, including PSC (Figure 2). With the normal values between 70 and 130% for stimulatory antibodies previously defined by ROC curves, we reached in the present study a specificity of 90%.

I.e., inhibitory antibodies against this receptor were found in immunoglobulin fractions from 49% of 63 PBC-patients, but only in 16% of 50 healthy controls (*P* < 0.001; Table 1) or in 20% of patients with PSC (*P* < 0.01). In patients with other liver disorders, these antibodies were detected in up to 26%. Stimulatory antibodies were found in only four PBC patients and one healthy control (Table 1). These data were obtained with a 1:100 dilution of immunoglobulins which had been shown to be optimal in previous studies (26). Higher immunoglobulin concentration did not increase the incidence of inhibitory antibodies but lead to stronger variations within the quadruplicate determinations for each sample and a reduced difference between the control with non-transfected cells and transfected cells (data not shown).

**Table 1:** Incidence of inhibitory or stimulatory effects of immunoglobulins from patients with different disorders on CHO/G5A-cells transfected with the mAChR3 plasmid.

**Diagnosis**	**Number patients tested**	**Effect on mAChR3-activity**		
		**None**	**Stimulatory**	**Inhibitory**
		**Number (%)**		
PBC	63	28 (45)	4 (6)	31 (49)^*^
PSC	50	40 (80)	0	10 (20)
Viral hepatitis	50	45 (90)	0	5 (10)
Alcoholic liver disease	50	37 (74)	0	13 (26)
Healthy controls	50	41 (82)	1 (2)	8 (16)

*Parenthesis reflects % patients' sera showing none, stimulatory, or inhibitory anti-mAChR3 reactivity*.

* *Significant as compared to patients with PSC (P < 0.01), viral hepatitis (P < 0.0001), alcoholic liver disease (P < 0.05), and healthy controls (P < 0.001)*.

### Prevalence of Functional Antibodies to the mAChR3 on CHO/G5A-Cells in the Course of the Disease in PBC Patients in Relation to the Therapy

In 38 PBC patients, the anti-AChR3 activity was investigated during the course of the disease. Of these patients, 10 (26%) had inhibitory, and 4 (11%) stimulatory antibodies.

**Figure 3:**
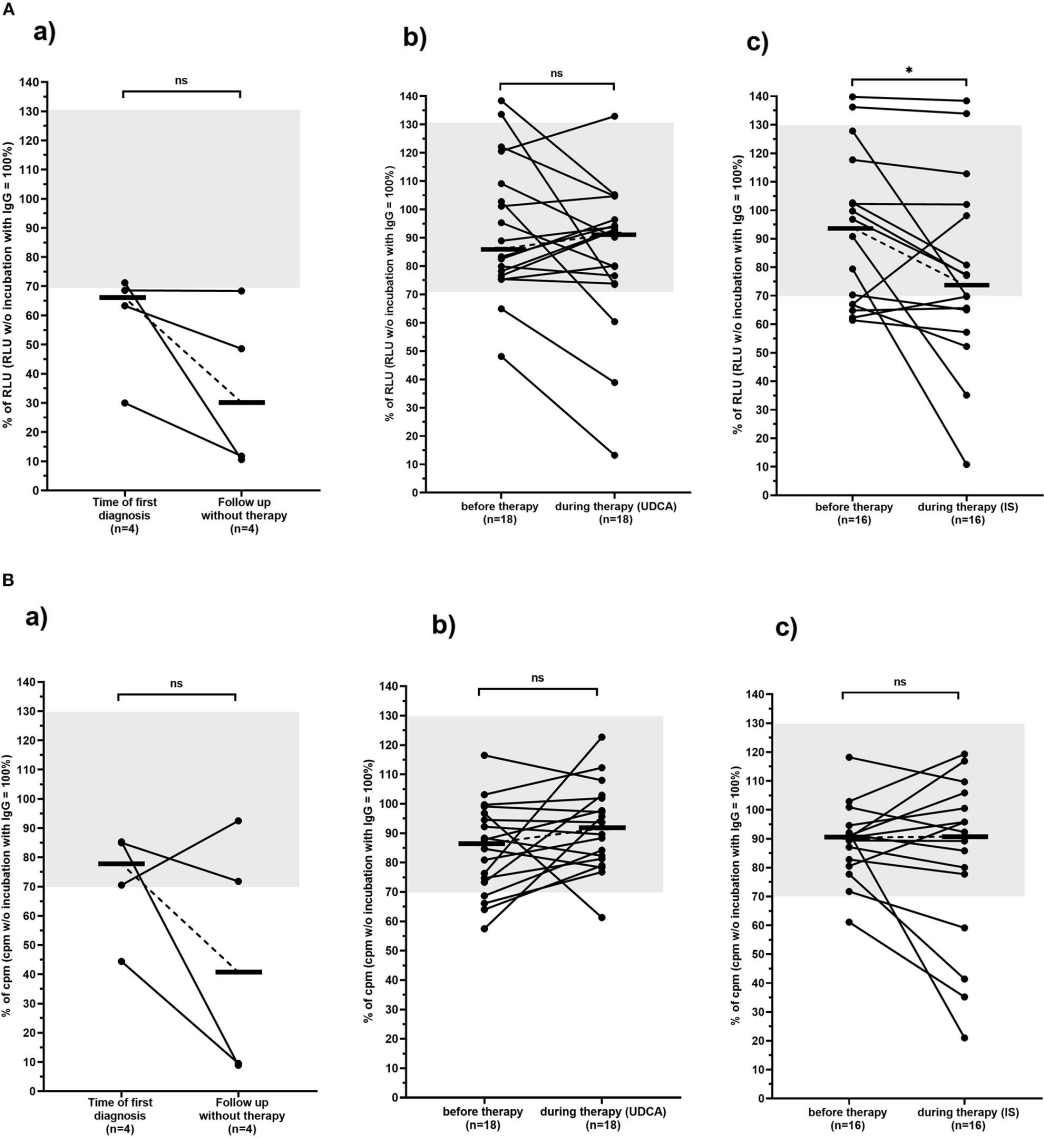
Carbachol-induced [Ca^2+^] signal in the mAChR3 functional assay **(A)** and proliferation **(B)** of CHO/G5A cells overexpressing mAChR3 after incubation of immunoglobulins from PBC patients before therapy and in the follow-up (a) without any therapy, (b) during therapy with UDCA, and (c) during therapy with immunosuppressive therapy. Data are given as percentage of the RLUs **(A)** or counts per minute (cpm) **(B)** w/o incubation with immunoglobulins. –, median; gray, normal range; n.s., not significant; *significant decrease (*P* < 0.05).

Four of the thirty-eight patients did not receive any therapy; 3 of them (75%) had inhibitory antibodies at the time point of first diagnosis. At the second time point (after 9–177 months; median, 103 months) all 4 (100%) had inhibitory antibodies (Table 2A, Figure 3A).

**Table 2A:** Effect of immunoglobulins from 38 PBC patients on mAChR3-activity on CHO/G5A-cells transfected with the mAChR3 plasmid before therapy and in the course without or with therapy.

**PBC patients**		**Effect on mAChR3-activity**		
		**None**	**Stimulatory**	**Inhibitory**
		**Number (%)**		
Without therapy (*n* = 4)	Time point of diagnosis	1 (25)	0	3 (75)
	After 9–177 months (median 103 months)	0	0	4 (100)
UDCA-therapy (*n* = 18)	Before therapy	14 (79)	2 (11)	2 (11)
	Under therapy (12–206 months, median 121 months)	14 (78)	1 (6)	3 (17)^*^
Immunosuppressive therapy (*n* = 16)	Before therapy	9 (56)	2 (13)	5 (31)
	Under therapy (11–213 months, median 108 months)	6 (36)	2 (13)	8 (50)

*Parenthesis reflects % patients' sera showing none, stimulatory, or inhibitory anti-mAChR3 reactivity*.

* *Significantly lower than in patients without therapy (p < 0.01) and in patients with immunosuppressive therapy (p < 0.05)*.

Of the 18 patients treated with UDCA, 2 (11%) had inhibitory and 2 (11%) stimulatory antibodies to the mAChR3 before therapy. In the follow-up, for 12–206 months (median, 121 months) 3 patients (17%) had inhibitory, and 1 had (6%) stimulatory antibodies (Table 2A, Figure 3A). The changes were not significant.

Five of the sixteen patients (31%) who received immunosuppressive therapy (with or without UDCA) showed inhibitory antibodies, and 2 (13%) showed stimulatory antibodies before therapy (Table 2A, Figure 3A). In the follow-up, 8 (50%) and 2 patients (13%), respectively, were positive. Although the increase of prevalence of inhibitory antibodies was not significant, there was a significant decline in receptor activity during therapy (Figure 3B; *P* < 0.05).

### Influence of Immunoglobulins From PBC Patients on CHO/G5A Cell Proliferation

The influence of patients' immunoglobulins on the proliferation of mAChR3-transfected CHO/G5A cells was investigated with the ^3^H-thymidine uptake test in the group of 38 PBC patients (Table 2B, Figure 3B).

**Table 2B:** Effect of immunoglobulins from 38 PBC patients on proliferation of mAChR3-transfected CHO/G5A-cells before therapy and in the course without or with therapy.

**PBC patients**		**Effect on proliferation**		
		**None**	**Stimulatory**	**Inhibitory**
		**Number (%)**		
Without therapy (*n* = 4)	Time point of diagnosis	3 (75)	0	1 (25)
	After 9–177 months (median 103 months)	2 (50)	0	2 (50)
UDCA-therapy (*n* = 18)	Before therapy	14 (78)	0	4 (22)
	Under therapy (12–206 months, median 121 months)	17 (94)	0	1 (6)
Immunosuppressive therapy (*n* = 16)	Before therapy	15 (94)	0	1 (6)
	Under therapy (11–213 months, median 108 months)	12 (75)	0	4 (25)

*Parenthesis reflects % patients' sera showing none, stimulatory, or inhibitory anti-mAChR3 reactivity. There were no significant differences between any of the groups*.

Immunoglobulins from 6 patients (16%) inhibited cell proliferation at time of first diagnosis, 7 (21%) in the follow-up.

Of the 4 non-treated PBC patients, 1 (25%) had inhibitory immunoglobulins at time of diagnosis; after an observation period of 9–177 months, they were observed in 2 patients.

Of the 18 PBC patients treated with UDCA, 4 (22%) had immunoglobulins inhibiting cell proliferation before therapy. Interestingly in all 4 patients this inhibitory property disappeared during UDCA therapy (12–206 months). However, in 1 patient, inhibitory activity newly developed during therapy (Table 2B, Figure 3B).

In contrast, within the group of patients treated with UDCA in combination with immunosuppressant agents (11–213 months), 3 newly developed antibodies inhibiting cell proliferation (Table 2B, Figure 3B).

In general, the behavior of the antibodies during therapy was rather heterogeneous, and there was no correlation with clinical parameters and response to therapy.

### Correlation Between mAChR3 Activity and Cell Proliferation Using CHO/G5A Cells

There was no correlation between influence of the immunoglobulins on the mAChR3 activity measured in the luminometric assay and the effect on the proliferation of mAChR3-transfected CHO-G5A cells measured in the ^3^H-thymidine uptake test (data not shown).

### Reactivity of Immunoglobulins From PBC Patients With TFK-1 Cells Constitutively Expressing mAChR3

In the next step, we analyzed the effect of antibodies of PBC patients on the mAChR3 receptor on cholangiocytes. Immunoglobulins from the 38 PBC patients before and during therapy were, therefore, applied in the functional mAChR3 assay and the proliferation assay using TFK-1 cells.

Immunoglobulins from 30 of the 38 PBC patients (79%) inhibited mAChR3 function on TFK-1 cells; 1 patient had stimulatory antibodies, i.e., inhibitory anti-mAChR3 antibodies were more frequently detected using cholangiocytes as substrate as, compared to mAChR3-transfected CHO/G5A-cells (79 vs. 26%; *P* < 0.0001). This number was independent from the dilution of immunoglobulins. The reactivity of the anti-mAChR3 antibodies did not differ significantly within the three treatment groups, neither before therapy nor at the end of the observation period during therapy (*P* > 0.05).

**Figure 4:**
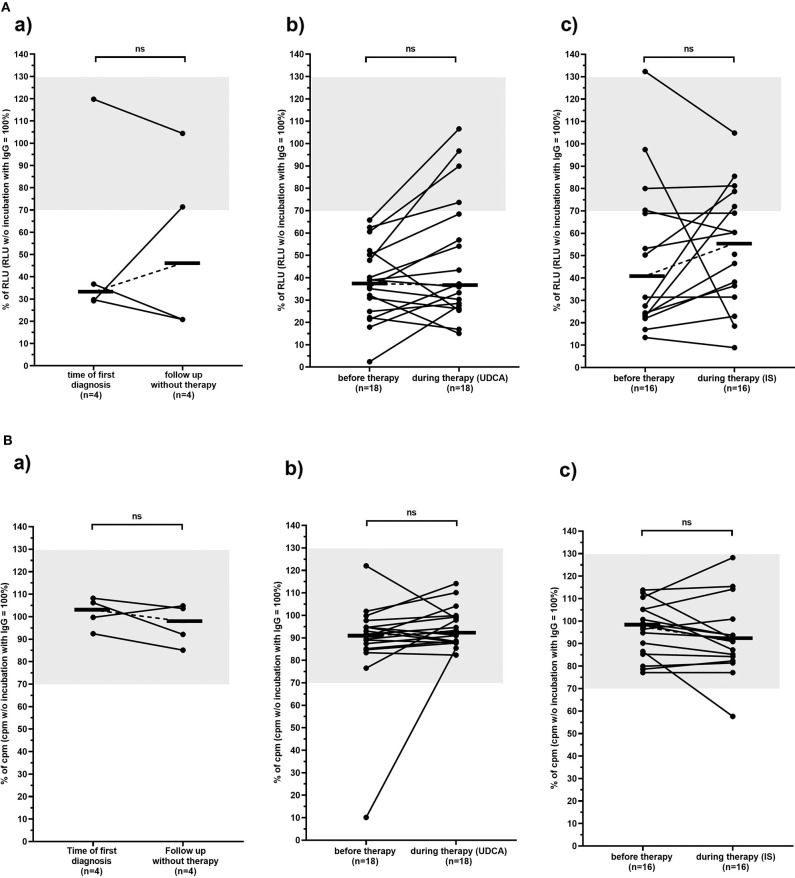
Carbachol-induced [Ca^2+^] signal in the mAchR3 functional assay **(A)** and proliferation **(B)** of cholangiocytes (TFK-1 cells) constitutively expressing mAChR3 after incubation of immunoglobulins from PBC patients before therapy and in the follow-up (a) without any therapy, (b) during therapy with UDCA, (c) during therapy with immunosuppressive therapy. Data are given as percentage of RLUs **(A)** or counts per minute (cpm) **(B)** w/o incubation with immunoglobulins. –, median; gray, normal range; n.s., not significant.

As shown in Table 3A, the incidence of anti-mAChR3 antibodies slightly decreased during treatment with UDCA but hardly changed in patients without therapy or during immunosuppressive treatment. There was no significant difference in the effect of antibodies on mAChR3-activity in all three treatment groups (Figure 4A).

**Table 3A:** Effect of immunoglobulins from 38 PBC patients on mAChR3-activity on cholangiocytes (TFK-1-cells) before therapy and in the course without or with therapy.

**PBC patients**		**Effect on mAChR3-activity**		
		**None**	**Stimulatory**	**Inhibitory**
		**Number (%)**		
Without therapy (*n* = 4)	Time point of diagnosis	3 (75)	0	1 (25)
	After 9–177 months (median 103 months)	2 (50)	0	2 (50)
UDCA-therapy (*n* = 18)	Before therapy	0	0	18 (100)
	Under therapy (12–206 months, median 121 months)	4 (22)	0	14 (78)
Immunosuppressive therapy (*n* = 16)	Before therapy	3 (19)	2 (13)	11 (69)
	Under therapy (11–213 months, median 108 months)	5 (31)	0	11 (69)

*Parenthesis reflects % patients' sera showing none, stimulatory, or inhibitory anti-mAChR3 reactivity. There were no significant differences between any of the groups*.

In contrast to the CHO/G5A-cells, proliferation of TFK-1 cells was influenced by immunoglobulins in only 1 patient, and also, in the follow-up, no significant change was observed (Table 3B, Figure 4B).

**Table 3B:** Effect of immunoglobulins from 38 PBC patients on proliferation of mAChR3-expressing cholangiocytes before therapy and in the course without or with therapy.

**PBC patients**		**Effect on proliferation**		
		**None**	**Stimulatory**	**Inhibitory**
		**Number (%)**		
Without therapy (*n* = 4)	Time point of diagnosis	4 (100)	0	0
	After 9–177 months (median 103 months)	4 (100)	0	0
UDCA-therapy (*n* = 18)	Before therapy	17 (94)	0	1 (1)
	Under therapy (12–206 months, median 121 months)	18 (100)	0	0
Immunosuppressive therapy (*n* = 16)	Before therapy	16 (100)	0	0
	Under therapy (11–213 months, median 108 months)	15 (94)	0	1 (1)

*Parenthesis reflects % patients' sera showing none, stimulatory, or inhibitory anti-mAChR3 reactivity. There were no significant differences between any of the groups*.

Again, there was no correlation between the effect of patients' immunoglobulins on the mAChR3 function and proliferative response of TFK-1 cells.

### Comparison of the Anti-mAChR3 Reactivity With the Presence of Anti-M2/PDC-E2 Antibodies

For the demonstration of anti-M2/PDC-E2 antibodies, there was no difference comparing the reactivity of patients' sera and purified immunoglobulins.

Of the 38 PBC patients 32 (84%) had anti-mAChR3 and anti-M2/PDC-E2 antibodies (of the IgG- and/or IgM-type) in parallel (Table 4). Four had only anti-M2/PDC-E2 antibodies, and 2 had only anti-mAChR3 antibodies. These were positive for other PBC-specific antibodies, i.e., antibodies to sp100 (*n* = 1) and gp210 (*n* = 1).

**Table 4:** Correlation between the presence of functional antibodies to the mAChR3 on TFK-cells and anti-M2 antibodies of the IgG- and IgM-type by ELISA.

**Antibodies to**			**Number (percent) patients positive**
**mAChR3-TFK-1**	**M2 (IgG-type)**	**M2 (IgM-type)**	
Positive	Positive	Positive	16 (42)
Positive	Positive	Negative	11 (29)
Positive	Negative	Positive	5 (13)
Positive	Negative	Negative	2 (5)^*^
Negative	Positive	Positive	2 (5)
Negative	Positive	Negative	2 (5)

*Parenthesis reflects % patients' sera showing none, stimulatory, or inhibitory anti-mAChR3 reactivity. All 36 anti-M2 positive sera reacted with the PDC-E2*.

* *Of these two anti-M2 negative patients one had antibodies to nuclear dots (sp100) and one to gp210*.

There was no correlation between the reactivity of anti-mAChR3- and anti-M2/PDC-E2 antibodies of the IgG (*r* = −0.02) or IgM-type (*r* = 0.02).

Similarly to the anti-mAChR3 antibodies, the reactivity of anti-M2/PDC-E2 antibodies hardly changed during UDCA or immunosuppressive therapy.

### Correlation Between Anti-mAChR3 Reactivity and Clinical Parameters

There was no correlation between the reactivity of antibodies to the mAChR3 expressed either by CHO-G5A- or TFK-1 cells and any laboratory parameters (alkaline phosphatase, transaminases, bilirubin, immunoglobulins, cholinesterase etc.). Moreover, no association with clinical symptoms such as Sicca symptoms, pruritus or arthralgia was observed. There was also no significant difference in antibody reactivity comparing patients with early PBC (stage I/II; %RLU 44.5 ± 30.1) and late stages of PBC (stage III/IV; % RLU 57.6 ± 42.4; *P* = 0.38).

**Figure 5:**
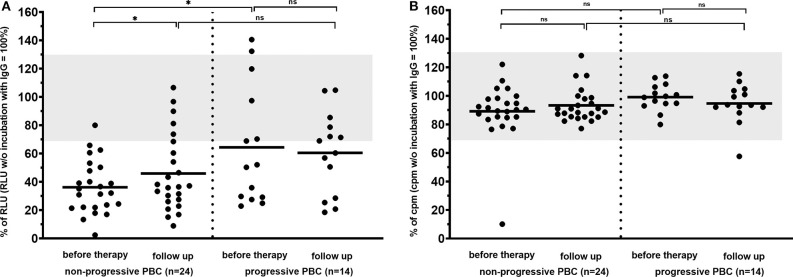
Carbachol-induced [Ca^2+^] signal in the mAChR3 functional assay **(A)** and proliferation **(B)** of cholangiocytes (TFK-1 cells) constitutively expressing mAChR3 after incubation of immunoglobulins from PBC patients with PBC at time of first diagnosis and in the follow-up. Data are given as percentage of the RLUs (a) or counts per minute (cpm) (b) w/o incubation with immunoglobulins. –, median; gray, normal range; n.s., not significant; *significant *P* < 0.05.

However, dividing the patients into those with an inactive benign (*n* = 24) and an active progressive course (*n* = 14) of the disease, inhibitory antibodies to the mAChR3 on TFK-1 cells were significantly more prevalent at time of first diagnosis in the former group (96%) than in patients with progressive PBC (57%; *P* < 0.01). Also, the inhibitory activity of antibodies was significantly stronger in patients with non-progressive as compared to patients with progressive PBC (Figure 5). In the follow-up (with or without therapy), 4 patients of the non-progressive group, but none of the patients of the progressive group, lost their inhibitory antibodies. Interestingly, stimulatory antibodies were found only in patients with progressive PBC.

Interestingly, the patient in the untreated group who lost inhibitory antibody reactivity in the course showed a progressive course (Figure 4A). In the UDCA-treated patients as well as in the IS-treated patients (Figure 4Aa–c), such an association was not observed. There was also no correlation between response to therapy (decrease of liver enzymes, immunoglobulins) and the course of antibody reactivity.

## Discussion

This is the first study showing that, in patients with PBC-functional antibodies to the muscarinic receptor M3 can be observed, thereby confirming our previous preliminary findings (25). These data were obtained with a luminometric assay, using CHO-cells transfected with the mAChR3 in the first approach. Thus, we found in 55% of PBC patients a change of receptor reactivity (49% inhibiting, 6% stimulating) of carbachol-stimulated mAChR3 expressed on CHO-cells, when purified immunoglobulins from patients' sera were added.

The application of purified immunoglobulin instead of whole serum is mandatory in those functional assays, in order to avoid non-specific reactivity of the mAChR3 receptor with other serum proteins. We used immunoglobulins purified by ammonium sulfate precipitation, because we showed in our previous study that it was superior to the isolation by Melon Gel IgG spin-purification, with respect to time for purification, costs, and even variations within the four-fold determination (30). Moreover, besides the immunoglobulins, we did not detect any other serum proteins by gel electrophoreses, and this was proven in the present study.

The prevalence of inhibitory antibodies was significantly higher in PBC than in controls including PSC. However, the antibodies did not correlate with clinical symptoms (Sicca syndrome, pruritus, and fatigue), laboratory parameters, histological stages, or relapse of the disease. Moreover, antibody reactivity hardly changed in the follow-up for up to 20 years, independently of kind of therapy.

Considering the fact that mAChR3 receptors are also expressed by cholangiocytes at the basolateral domain (20, 39), we applied in a second approach TFK-1 cells, a cholangiocyte cell line, to the luminometric assay. Indeed, we could confirm that these cells express the mAChR3 already constitutively. Prevalence of inhibitory antibodies in this system was even higher than with CHO-cells (79 vs. 49%). The incidence of reactivity toward CHO-cells could not be increased by applying higher or lower immunoglobulin concentrations in the assay. Interestingly, analyzing immunoglobulins from patients with Sjoegren syndrome, we observed an opposite effect; thus, 50% contained inhibitory antibodies to the mAChR3 expressed on CHO-cells, but only 14% to the mAChR3-expressing TFK-cells (own unpublished observation). This probably indicates that the differences in reactivity toward the human mAChR3 on CHO-cells or cholangiocytes may not depend upon the used immunoglobulin concentrations or different expression levels of the mAChR3 on these cell lines but rather some kind of disease-, “organ”-, or “species”-specificity; i.e., anti-mAChR3 antibodies in different disorders presumably recognize different molecular forms of human mAChR3 expressed on the transfected animal-derived ovary cell line, as compared with the human cholangiocytic cell line originating from bile duct carcinoma, and this may lead to different intracellular signals.

In most patients, antibodies to the mAChR3 and M2/PDC-E2 occurred simultaneously. However, there were 4 patients with strong anti-M2/PDC-E2 reactivity, but no anti-mAChR3 reactivity, and 2 were anti-mAChR3 positive, but anti-M2/PDC-E2 negative, i.e., the antibodies may be present also in anti-M2 negative patients. In the latter 2 patients, diagnosis of PBC had been confirmed by histological analysis, and both of them had other PBC-specific antibodies, i.e., antibodies to nuclear dots (sp100) and nuclear membrane (gp210). Similarly to the anti-mAChR3 antibodies, the anti-M2 antibody reactivity hardly changed during the course of the disease (data not shown), as already known from previous studies (36, 40, 41). Despite the high co-occurrence of anti-mAChR3 and anti-M2/PDC-2 a cross-reactivity can be excluded with high probability since there was no correlation between their antibody reactivity in the luminometric assay and ELISA. Moreover, absorption of patients' immunoglobulins with the M2-antigen did not influence their reactivity with the mAChR3 (own unpublished observation).

Also, the antibodies inhibiting mAChR3 reactivity on TFK-1 cells in the presented PBC patients did not correlate with clinical, histological or laboratory parameters. However, our preliminary data indicate that there might be an association with disease activity. Thus, patients with a rather benign course being in stage I/II without progression to late stages within an observation period of up to 10 years had a significantly stronger inhibitory activity on M3R-expressing TFK-1 cells at time of first diagnosis than the inhibitory activity of patients with a progressive course, as defined by the development of liver failure during that time. Recent studies indicate that acetylcholine plays a significant permissive role in sustaining cholangiocyte reaction to cholestasis as far as survival is concerned. Interruption of the cholinergic innervation by vagotomy induces a marked decrease in total bile duct mass caused by impaired cholangiocyte proliferative capacity and intracellular cAMP levels and enhanced cell death by apoptosis (42–44); i.e., cholangiocyte biology is under the regulation of visceral hormones, neuropeptides, and neurotransmission. Activation of basolateral mAChR3 by acetylcholine induces an increase of intracellular [Ca^2+^] concentration via an increase of intracellular inositol triphosphate concentration. This leads via activation of cAMP and of the CaCl-transport to an increased release of Cl- and by an activation of anion exchanger 2 to a secretion of bicarbonate (HCO3-) into the bile duct lumen (20). Thus, it seems not unlikely that alterations in mAChR3-coupled signal transduction pathways may contribute to the pathogenesis of chronic-inflammatory bile duct disorders. This may also implicate an alteration of the “biliary HCO3-umbrella,” as postulated by Beuers et al. and Hohenester et al. by inhibition of HCO3-secretion (45, 46). Indeed, synthetic mAChR3 blockade in intestinal epithelial cells in the mouse in early stages decreased HCO3-release (47). But also dysregulation of mAChR3-induced signal transduction pathways could be involved in the pathogenesis of chronic inflammatory disorders by an alteration of the protective barrier function of bile duct epithelial cells. In this respect, it is of interest that the T allele of the receptor is overrepresented in PBC, as compared to controls, probably due to a cholinergic receptor muscarinic 3 (*CHRM3*) SNP rs4620530 polymorphism in these patients, and it was hypothesized that it may be a potential risk for the development of PBC (48).

In PBC, cholangiocytes show enhanced proliferative activities together with an increased rate of apoptosis (49, 50). Its course is characterized by a balance between cholangiocyte death and compensatory cholangiocyte proliferation (51). Acetylcholine increases cholangiocyte proliferation via [Ca^2+^] activation and calcineurin-mediated positive modulation of adenylate cyclase with enhanced cAMP intracellular levels (42). Inhibitory antibodies may have a protective function by reducing cholangiocyte proliferation, fitting with our observation that they occur preferentially in patients with a more benign course of PBC. One of our four patients without therapy lost his inhibitory anti-mAChR3 antibodies in the course of the disease, and this was the only one in this group who had a progressive course. A shift of antigen specificity toward distinct parts of the receptor could go along with a loss of protection and possibly tissue damage. In a mouse model for chronic-inflammatory bile duct disorders the genetic loss of mAChR3 was, indeed, associated with a worse outcome (52).

In general, the response of anti-mAChR3 antibody reactivity to therapy was only marginal with large inter-individual differences, as already described for anti-M2/PDC-2 antibodies. We also did not find a correlation between antibody reactivity and response to therapy. However, we do not yet know whether other external or internal factors may affect antibody reactivity in individual patients. Also, in patients with Sjoegren syndrome, therapy did not influence anti-mAChR3 reactivity (own unpublished observation).

Although there was a clear effect of the antibodies on mAChR3-function, proliferation of the TFK-cells was hardly influenced. However, it has been shown that only proliferating, but not quiescent cholangiocytes, are influenced in their functions by neuropeptides and neurotransmitters. In our “model,” we used cell lines from tumors that may be already altered in their proliferative response, and this may explain why we observed an inhibition of the mAChR3 by antibodies in PBC sera, but no effect on the proliferative response. In this respect, cholangiocytes isolated from biopsies of PBC patients might be of interest. The existence of functional autoantibodies in PBC interfering with neurotransmitters and their receptors with transporter molecules or with apoptosis molecules would possibly provide a link between autoimmunity and cholangiocyte alteration in PBC (53).

In the present study, we found that immunoglobulins derived from patients' sera inhibited the carbachol-induced M3R activity on CHO- or TFK-1 cells, i.e., they have an antagonistic effect. Data in the literature are rather inconclusive showing either agonistic or antagonistic effects of patients' sera (10, 27, 32, 33, 54, 55). Differences in experimental conditions, especially with regard to the systems used to demonstrate binding of anti-M3R to the muscarinic receptors, may play a major role for those discrepancies (9), as already known from the demonstration of antibodies to the nicotinic receptor in myasthenia gravis (2). Furthermore, differences in autoantibody titers, isotypes of autoantibodies, duration, and severity of the patients' disease states may play a role. It was also shown that Sjoegren syndrome-like disease may be dependent on anti-M3R autoantibodies of a specific isotype, i.e., IgG1.

In conclusion, we have shown for the first time that functionally active autoantibodies toward the mAChR3 occur in sera from patients with PBC, and that they are associated predominantly with a clinically inactive course suggesting a protective role. However, this has to be proven in larger studies. Moreover, it still remains to be seen whether this *in vitro* phenomenon may play any role also *in vivo*.

## Data Availability Statement

The datasets generated for this study are available on request to the corresponding author.

## Ethics Statement

The studies involving human participants were reviewed and approved by Ethik-Kommission an der Medizinischen Fakultät der Eberhard-Karls-Universität Tübingen und am Universitätsklinikum Tübingen. The patients/participants provided their written informed consent to participate in this study.

## Author Contributions

CB and RK designed and coordinated the study. CB and JG provided patients' sera and clinical data. CM and BP performed the experiments, acquired, and analyzed data. BP, CM, and RK interpreted the data. RK and BP wrote the manuscript. All authors approved the final version of the article and agreed to publication.

## Conflict of Interest

The authors declare that the research was conducted in the absence of any commercial or financial relationships that could be construed as a potential conflict of interest.
